# Niche switching and leapfrog foraging: movement ecology of sympatric petrels during the early breeding season

**DOI:** 10.1186/s40462-020-00212-y

**Published:** 2020-05-29

**Authors:** Petra Quillfeldt, Henri Weimerskirch, Karine Delord, Yves Cherel

**Affiliations:** 1 Department of Animal Ecology and Systematics, Justus Liebig University Giessen, Heinrich-Buff-Ring 26, 35392 Giessen, Germany; 2 Centre d’Etudes Biologiques de Chizé, UMR 7372 du CNRS-La Rochelle Université, 79360 Villiers-en-Bois, France

**Keywords:** Breeding schedule, Central-place forager, Foraging ecology, Tracking

## Abstract

**Background:**

The timing of events in the early part of the breeding season is crucially important for successful reproduction. Long-lived animals that migrate large distances independently of each other meet at the breeding sites to re-establish their pair bonds and coordinate their breeding duties with their partners.

**Methods:**

Using miniature light-geolocation and immersion data together with blood stable isotopes, we studied the early breeding season in Thin-billed prions *Pachyptila belcheri,* Antarctic prions *P. desolata* and Blue petrels *Halobaena caerulea* breeding at Kerguelen Islands in the Indian Ocean. These three species exhibit differences in their winter habitat and timing of migration, moult and breeding. We hypothesised that these differences would influence their behaviour during the early breeding season.

**Results:**

In line with our hypothesis, we found clear differences not only in the timing of colony attendance, but also in the time budgets while at sea and in habitat use. Both early breeding Blue petrels and late breeding Antarctic prions spent about 8 h per day in flight and 15 h foraging. In comparison, Thin-billed prions, which breed in mid-summer, spent less time (5 h daily) in flight and more time (18 h daily) foraging, thus maximizing the time spent foraging during the longest daylight days of the year. While the ecological habitat parameters (sea temperature, wind, productivity) of Thin-billed prions and Blue petrels were relatively stable throughout the year, Antarctic prions showed clear niche switching, caused by leapfrogging between the northernmost winter distribution to the southernmost distribution during the early breeding season. Blood stable isotopes confirmed the habitat switch between the inter-breeding and early breeding periods and highlighted trophic segregation with Blue petrels feeding more on fish and Antarctic petrels more on crustaceans during the early breeding period.

**Conclusion:**

We found that the three sympatric petrel species segregated in time and space, both in the winter and the early breeding season. The interplay of timing and distribution meant that the three species show the full range of migratory strategies, from niche-tracking Blue petrels to niche-switching Antarctic prions. The latitudinal distribution resembled the leapfrogging of terrestrial avian migrant species or populations.

## Introduction

Animals often aggregate in large numbers at rich foraging grounds and suitable breeding sites. This aggregation may lead to intense competition for resources [1] and reduced foraging efficiency [2]. Interspecific competition can be reduced by ecological segregation [3] and according to the niche theory, segregation in some dimension of the n-dimensional niche hyper-volume between sympatric species is essential for their coexistence in sympatry (e.g., [4–6]). Niche differentiation thus separates sympatric species and promotes coexistence of ecologically similar species via their segregation.

Possible dimensions of segregation include habitat, resource, and temporal axes [7]. Habitat segregation can be seen as differences in foraging areas [8, 9] or diving depths [9, 10], and species differences in prey choice (e.g. [11, 12] arise from resource segregation. Differences at temporal axes such as in the timing of breeding may also be important, because they determine the time of the highest energy demand in each species in relation to the accessibility of prey. In addition to the timing of events within the year, the daily timing of foraging may also be crucial, for example if nocturnal seabirds take advantage of the daily vertical migration of marine organisms.

A good example for aggregated animals can be found in seabirds that often aggregate in large numbers at suitable breeding colonies on oceanic islands and at other protected sites. In a previous study, we investigated spatial segregation in the winter distribution of closely related petrel species breeding sympatrically in the southern Indian Ocean [13], as these highly mobile petrels offer fine models of evolution of diversity along environmental gradients. The Blue petrel (*Halobaena caerulea*, BP) is the only member of the genus Halobaena, and the sister group to the prions (e.g. [14]). We found that Thin-billed prions (*Pachyptila belcheri*, TBP), Antarctic prions (*Pachyptila desolata*, AP) and blue petrels from the Kerguelen archipelago in the southern Indian Ocean segregated latitudinally: Antarctic prions spent the winter north of the Polar Front in temperate waters, whereas Thin-billed prions preferred intermediate latitudes and temperatures and blue petrels were found south of the Polar Front in Antarctic waters. This near complete niche separation across a large-scale ecological gradient without physical barriers was also reflected in stable isotope values of feathers, suggesting evolutionary isolation by environment.

The aim of the present study was to investigate ecological segregation of Antarctic prions, Thin-billed prions and Blue petrels during the early part of the breeding season, from the arrival at the colony to the first incubation shift. In addition to winter habitat choice, these species differ in their timing of migration and molt of flight feathers [15]. While Blue petrels and Thin-billed prions molt during the post-breeding period in polar waters, Antarctic prions molt several months later, in the pre-breeding period, and further north, in warm subtropical waters [15].

We hypothesised that these differences, and the different environmental conditions experienced during the winter, would influence their behaviour during the early breeding season. In the present study, we using bio-logging data to: (i) compare colony attendance patterns among the three species, (ii) determine the foraging areas used during the pre-laying exodus and early incubation, and (iii) examine the activity data of tracked birds during the early breeding season. We also investigated the isotopic niche of the equipped birds during the early breeding season by measuring blood δ^13^C and δ^15^N values as proxies of their foraging habitat and diet/trophic position, respectively [16].

## Materials and methods

### Fieldwork, study species and data loggers

Fieldwork was carried out at the subantarctic Kerguelen archipelago in 2011–2013. To investigate the timing and spatial extent of movements, we attached leg-mounted miniaturized saltwater immersion geolocators (MK10, developed by British Antarctic Survey, Cambridge, UK) to breeding adults at two adjacent islands in the Golfe du Morbihan, Ile Mayes (49°28′S, 69°57′E) and Ile Verte (49°31′S, 70°04′E), where large numbers of BP and TBP, and of AP breed, respectively (for sample sizes, see Table 1). Nests were selected according to accessibility, and the birds were captured by hand at the nests during incubation. The geolocators weighed 1 g (< 1% of the mean body mass) and were fixed to plastic leg bands. Tagged individuals were marked with numbered steel rings on the other leg. A blood sample (0.1 ml) for sex determination was taken from the brachial vein and stored on FTA cards. Burrows were revisited and devices retrieved during early incubation in the following season (Table 1), and birds were blood sampled for stable isotope measurements (0.2–0.4 ml from the brachial vein). All birds were equipped with geolocators during the 2011/2012 breeding season and the data in this study correspond to the beginning of the 2012/2013 breeding season. Because several loggers stopped recording several months before device recovery, the final sample sizes for year-round tracks, which were used in this study, were smaller than for recovered data sets. A detailed study found no evidence for any substantial impact of the geolocators on Thin-billed prions: breeding performance was unaffected in the season of attachment or following recovery; eco-physiological measurements suggested that adults adapted to the higher load; and the similarity in stable isotope ratios in blood and feathers of instrumented adults and controls indicated that general diet and distribution was unaffected [17].

**Table 1:** Geolocator deployment and recovery and sample sizes for Blue petrels *Halobaena caerulea*, Thin-billed prions *Pachyptila belcheri* and Antarctic prions *P. desolata*, from Kerguelen Islands, Indian Ocean

Species	GLS		Tracks		Sex	
	Deployment	Recovery	All	Complete	Females	Males
	n	n (%)	n	n	n	n
Blue petrel	20	17 (85)	16	12	8	4
Thin-billed prion	29	23 (79)	21	15	8	7
Antarctic prion	20	11 (55)	11	11	4	7

The breeding chronology and colony attendance vary among the species. The following features are notable: (i) fledging occurs in February in both BP and TBP, and later, in April, in AP; (ii) unlike prions, adult BP transiently return to their burrows in fall during a post-breeding visit [18, 19]; (iii) BP return to the colony in early September, while TBP and AP arrive later, in October and November, respectively; (iv) importantly, AP lays later than the two other species, with laying occurring on average 40 days later than in its closely-relative TBP [20].

The transition from the non-breeding to the breeding season starts with the re-occupation of the nest site, pair-bond re-establishment and mating. During this phase, Procellariiformes typically spend a high proportion of their time ashore, where they fast and lose weight [21]. To recover body condition and in the case of the females, to feed up for egg production, both male and female petrels often fly long distances to areas of high resource abundance during an extended foraging trip before laying - the pre-laying exodus or ‘honeymoon period’ [22]. As soon as the female returns from the pre-laying exodus, she lays a single egg, and departs again, while the male usually takes the first long incubation shift [21].

### Sex determination

The sex of each bird in this study was determined through PCR using primers 2550 and 2718 that amplify sections of the sex-linked chromo-helicase-DNA binding (CHD) gene [23]. DNA was extracted from 50 μl blood using a Qiagen DNAEasy blood purification kit (Qiagen, Hilden, Germany). Each reaction was carried out in 25 μl, containing 10 ng template DNA, 1 × PCR buffer, 0.1 mM DNTPs, 2.5 mM MgCl2, 0.2 μM of each primer and 0.1 U Taq polymerase (Firepol, Soilis Biodyne, Tartu). Thermocycling consisted of an initial denaturation step of 2 min at 94 °C, followed by 35 cycles denaturation at 94 °C for 30 s, annealing at 54 °C for 30 s, extension at 72 °C for 1 min, and ended with two expansion steps of 42 °C for 1 min and 72 °C for 10 min. PCR products were visualised on a 2% agarose gel, with a single band at ~ 650 bp indicating a male, and two bands at ~ 450 and ~ 650 bp indicating a female.

### Data processing

Geolocators provide two positions per day based on light levels, with an accuracy of approximately 186 ± 114 km [24]. Light data were analysed using the BASTrak software suite (British Antarctic Survey, Cambridge, UK). TransEdit was used to check for integrity of light curves and to determine dawn and dusk times, and Locator to estimate the latitude from day length and longitude from the time of local mid-day relative to Greenwich Mean Time. We assumed a sun elevation angle of − 3.5°, based on known positions obtained during pre- and post-deployment calibration of the loggers at the colony. All estimated locations were examined visually in a geographical information system (GIS) and any unrealistic positions – either associated with interference to light curves at dawn or dusk, or in proximity to equinoxes when latitudes are unreliable - were excluded from further analyses. We systematically excluded latitude position estimates during 1 week before and after the equinox, and any positions that were unrealistic during 2 weeks before and after the equinox.

Trips to sea were distinguished from periods in the burrow by examining the light and immersion data. Following [25], the occurrence of complete daytime darkness in the logger trace allowed identification of days spent in the colony, whilst sustained periods of night-time dryness in the immersion data allowed identification of visits to the burrows during the night. This allowed determining the day (or night) of first arrival in the colony arrival to the colony, which was followed by the “pre-exodus phase”, i.e. the period from first arrival to the start of the pre-laying exodus. During this phase, we determined the total number and proportion of days spent in the burrow. In addition to determining only days at the colony and days at sea (e.g. [26]), we here distinguished another category, namely days at sea followed by nights in the burrow. The pre-laying exodus was an obvious phase lasting 14–52 days when the bird was at sea, which preceded the first incubation shift. Days away from the nest during the incubation shifts indicated egg neglect (also termed intermittent incubation), a common strategy in petrels and some other offshore feeders (e.g. [27]).

The activity (i.e. saltwater contact) analyses were carried out with the online tool Actave.net [28] were used to describe the birds’ behaviour at sea. The devices recorded saltwater immersion every 3 s as proxy for activity patterns and store the sum of positive tests once every 10 min. Hence, each recorded time-stamped immersion value (denoted ε) can range from 0 (no immersion) to 200 (permanent immersion). Actave.net’s standard setting define immersion as follows (i) time in flight: the sum of all 10-min intervals with ε = 0 (dry), (ii) time (sitting) on water: the sum of 10-min intervals with ε = 200 (wet), and (iii) foraging time: the sum of 10-min intervals with 0 < ε < 200 (intermediate). Hence, the bird’s behaviour was defined using the less arbitrary and most conservative threshold ε values, namely 0 and 200. BP and prions use different feeding techniques, including surface-seizing, surface-filtering, shallow-plunging, hydroplaning and dipping [18]. These methods involve actively transitioning from air to water; accordingly, foraging time was here identified as intermittent wet and dry states for at least one 10 min period. Although such records probably include non-foraging behaviours (e.g. preening, stretching), we believe that they represent a reasonable indicator of foraging activity of the birds [29].

The following habitat parameters were obtained through the Environmental Data Automated Track Annotation System (Env-DATA) on Movebank (movebank.org): Daytime sea surface temperature (SST) and Chlorophyll a mass concentration near the surface of the ocean (ChlA) from MODIS Ocean Aqua OceanColor (4 km, 8 day mean), and the U and V wind components from ECMWF Interim Full Daily SFC Wind (10 m above ground). Wind speed was calculated from the square root of the sum of squares of the V (south to north) and U (west to east) wind components. We have previously shown that coarse-resolution location data are suitable for species distribution modelling despite an offset in the scale with the environmental data [30].

Statistical analyses were conducted using R 3.6.0 [31]. We tested for normality using Kolmogorov-Smirnov tests and by checking plots of the data. Throughout this study all means are given ± S.D. To compare habitat parameters a principal component analysis (PCA) of the five environmental parameters (SST, ChlA, windU, windV, windspeed) was performed. Factor analysis produces a reduced suite of independent dimensions for modelling, and thus helps to avoid overfitting [32]. The PCA extracted two significant components. PC1 (eigenvalue 1.70) was determined mainly by the U (west to east) wind component (*R* = -0.86) and wind speed (*R* = -0.88), and thus, was lower in stronger westerly winds. PC2 (eigenvalue 1.14) was determined mainly by ChlA (*R* = -0.76) and SST (*R* = 0.60) and thus, was lower in colder, more productive waters and higher in warmer, less productive waters. Habitat niches plots were created from the two dimensions of the habitat (PC1 and PC2) using kernel densities calculated in R 3.6.0 [31]. (Function kde2d in the R-package MASS).

### Stable isotope analyses

Carbon and nitrogen isotopic studies from the Southern Ocean show δ^13^C values of seabirds correspond to the location of their latitudinal foraging habitats [33, 34] and their δ^15^N values increase with trophic level [35]. Following [33], the δ^13^C estimations of the Subtropical and Polar fronts for blood were − 19.7 and − 22.5 ‰, respectively. From North to South they delineate the Subtropical (δ^13^C ≥ 19.7 ‰), Subantarctic (− 22.5 < δ^13^C < − 19.7 ‰) and Antarctic (δ^13^C ≤ 22.5 ‰) zones.

Whole blood collected during the retrieval of the geolocators was freeze-dried and one sub-sample in the order of 0.3–0.4 mg was weighed with a microbalance and packed in tin capsules. Carbon and nitrogen isotope ratios were measured simultaneously by continuous-flow isotope ratio mass spectrometry (Thermo Scientific DeltaV Advantage) coupled to an elemental analyser (Thermo Scientific Flash EA1112). Replicate measurements of internal laboratory standards indicated measurement errors < 0.10 ‰ for δ^13^C and δ^15^N. All stable isotope ratios are expressed in δ notation as parts per thousand (‰) deviation from the international standards Vienna-Pee Dee Belemnite (carbon) and AIR (nitrogen).

Whole blood δ^13^C and δ^15^N values are very close to those of blood cells because blood cells contain more organic matter than plasma [36]. Bird blood has a turnover time of ca. 4 weeks [37]. Thus, samples taken after recapture of the birds carrying GLS were representative of the early breeding season, in particular the pre-laying exodus. To complete the picture, blood δ^13^C and δ^15^N values were compared to those of body feathers that are indicative of the foraging ecology of the birds during the inter-breeding period [15]. Before comparison, feather values were corrected to take into account tissue-specific isotopic differences [38].

## Results

### Return to breeding colony

**Fig. 1:**
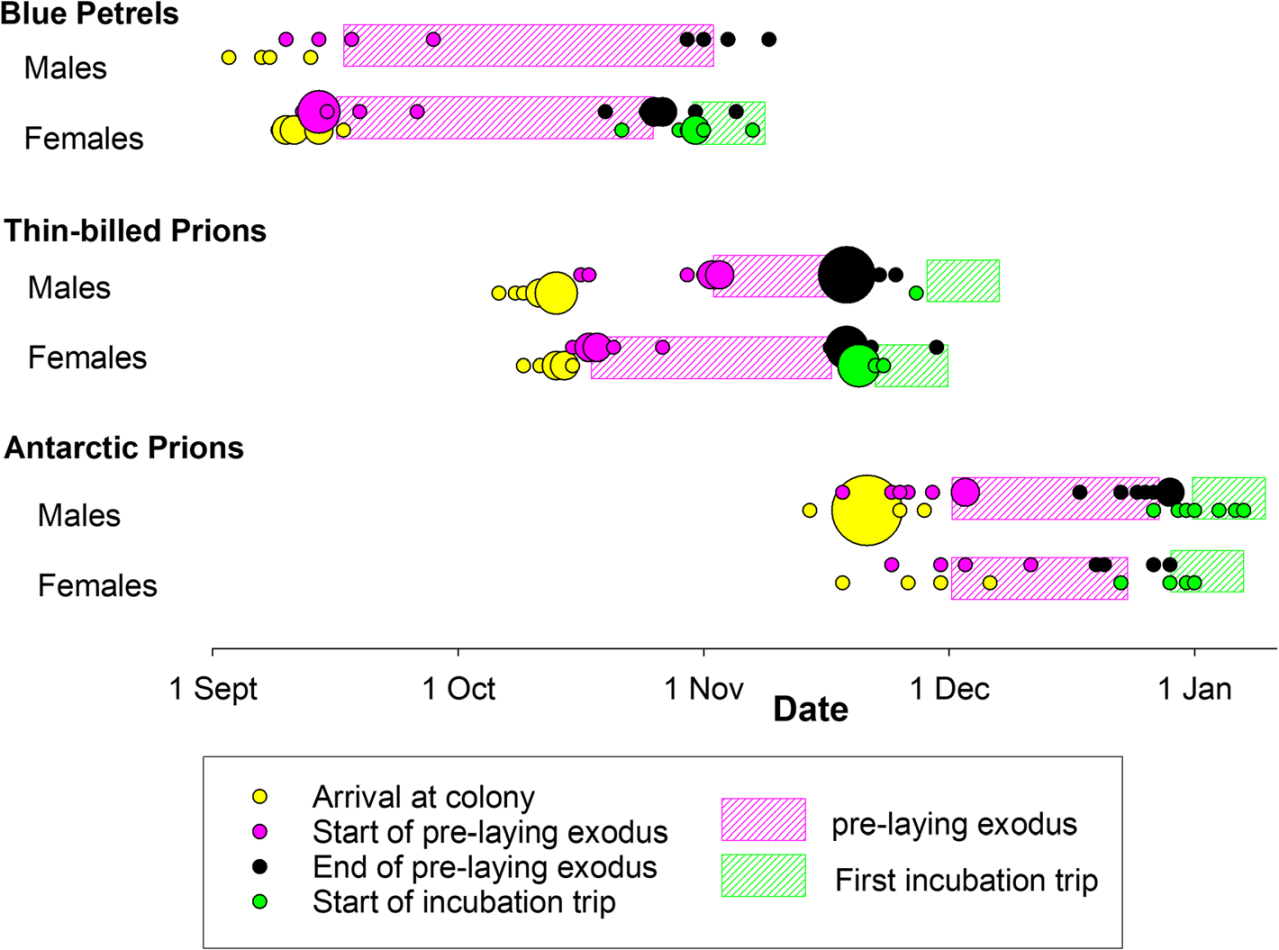
Early breeding season chronology of Blue Petrels, Thin-billed prions and Antarctic Prions from Kerguelen Islands. Larger dots mark more individuals starting or ending a phase on the same day. Note that Antarctic prions do not perform a continuous pre-laying exodus (e.g. see Electronic Supplement, Fig. S1), but the time bar here shows the length of the phase of alternating times at sea and in the colony

Arrival times back to Kerguelen (Fig. 1, Fig. S1, Table 2) varied among the species, with Blue petrels arriving in September, Thin-billed prions in October and Antarctic prions in November to early December (Table 3). Arrival dates also differed between sexes (Tables 2 and 3, Fig. 1, Fig S1), as males arrived earlier, on average.

**Table 2:** Early breeding season chronology of Blue Petrels, Thin-billed prions and Antarctic Prions from Kerguelen Islands

Dependent	Species	Sex	Species:Sex interaction
First arrival to colony	***F***_**2,32**_ **= 1043.4,*P*** **< 0.001**	***F***_**1,32**_ **= 9.5,*P*** **= 0.004**	*F*_2,32_ = 0.8, *P* = 0.460
Pre-exodus duration	***F***_**2,32**_ **= 405.5,*P*** **< 0.001**	***F***_**1,32**_ **= 6.2,*P*** **= 0.005**	***F***_**2,32**_ **= 3.4,*P*** **= 0.045**
Departure on exodus	***F***_**2,32**_ **= 402.2,*P*** **< 0.001**	*F*_1,32_ = 2.2, *P* = 0.150	***F***_**2,32**_ **= 4.7,*P*** **= 0.017**
Return from exodus	***F***_**2,32**_ **= 510.7,*P*** **< 0.001**	*F*_1,32_ = 6.8, *P* = 0.107	*F*_2,32_ = 2.7, *P* = 0.081
Exodus duration	***F***_**2,32**_ **= 29.2,*P*** **< 0.001**	*F*_1,32_ = 0.1, *P* = 0.749	***F***_**2,32**_ **= 7.1,*P*** **= 0.003**
Departure on incubation trip	***F***_**2,20**_ **= 341.9,*P*** **< 0.001**	***F***_**1,20**_ **= 5.1,*P*** **= 0.035**	*F*_2,20_ = 0.2, *P* = 0.668
Return from incubation trip	***F***_**2,20**_ **= 325.3,*P*** **< 0.001**	*F*_1,20_ = 2.7, *P* = 0.113	*F*_2,20_ < 0.1, *P* = 0.897
Incubation trip duration	***F***_**2,20**_ **= 10.9,*P*** **< 0.001**	*F*_1,20_ = 2.4, *P* = 0.137	*F*_2,20_ = 1.8, *P* = 0.200

Multifactorial ANOVA for the effects of species and sex on arrival dates and the timing and duration of the pre-laying exodus and the first incubation trip. Significant *p*-values are marked in bold

**Table 3:** Timing of the early breeding season phases in Blue Petrels, Thin-billed prions and Antarctic Prions from Kerguelen Islands

	Blue petrel		Thin-billed prion		Antarctic Prion	
	Males	Females	Males	Females	Males	Females
N	4	8	7	8	7	4
Arrival to the colony	8.9. (3.9.-15.9.)	12.9. (9.9.-19.9.)	10.10. (5.10.-12.10.)	12.10. (8.10.-14.10.)	20.11. (13.11.-27.11.)	27.11. (17.11.-5.12.)
Pre-exodus phase (days)	9.5 ± 6.4	3.9 ± 2.4	16.6 ± 9.0	5.1 ± 2.9	5.4 ± 2.2	4.5 ± 1.3
**Pre-laying exodus**						
Start	18.9. (11.9.-27.9.)	15.9. (11.9.-27.9.)	27.10. (15.10. − 1.11.)	20.10. (14.10.-25.10.)	26.11. (17.11.-2.12.)	1.12. (23.11.-10.12.)
End	3.11. (31.10.-9.11.)	29.10. (20.10.-15.11.)	18.11. (17.11.-23.11.)	19.11. (15.11.-26.11.)	23.12. (16.12.-27.12.)	22.12. (18.12.-27.12.)
Duration (days)	47.3 ± 3.2	44.1 ± 3.2	22.9 ± 7.5	32.1 ± 2.6	28.4 ± 5.3	22.2 ± 6.4
**First incubation trip: N**	0	7	1	6	7	4
Start	–	28.10. (20.10.-31.10.)	25.11. -	19.11. (17.11.-21.11.)	31.12. (25.12.-5.1.)	26.12. (21.12.-30.12.)
End	–	11.11. (2.11.-14.11.)	2.12. -	28.11. (27.11.-1.12.)	8.1. (3.1.-12.1.)	6.1. (4.1.-7.1.)
Duration (days)	–	14.4 ± 0.7	8	10.7 ± 0.7	8.7 ± 0.7	8.8 ± 2.0

Means and ranges are given for dates, and means and standard deviation for durations. Because Antarctic prions do not undertake one long pre-laying exodus, the total duration of the phase is given for this species

The pre-exodus phase (i.e. from first arrival at the colony to departure for the pre-laying exodus) lasted 3–27 days, with significant differences between the species and sexes (Tables 2 and 3, Fig. 1, Fig. S1). During the pre-exodus phase, birds spent shifts of 1–19 days in the burrow (Fig. 1, Fig. S1). In Thin-billed prions, which had the longest pre-exodus phase, burrow visits were alternated with foraging trips lasting 1–13 days (Fig. S1).

### Pre-laying exodus

Departure on exodus was in the same order as arrival, i.e. Blue petrels started in September, Thin-billed prions in October and Antarctic prions in November to early December (Fig. 1, Fig. S1, Table 3). We observed no consistent sex differences: Females departed slightly earlier than males in Blue petrels and Thin-billed prions, but the opposite was found in Antarctic prions (Table 3). Likewise, sex differences in the pre-laying exodus duration were species-specific (Tables 2 and 3): Females engaged in longer pre-laying exoduses than males in Thin-billed prions, while the opposite was observed in Blue petrels and Antarctic prions. Females also reached further distances to the colony (Table 4).

**Table 4:** Distribution and activity parameters of Blue Petrels, Thin-billed prions and Antarctic Prions from Kerguelen Islands during the pre-laying exodus

	Blue petrel		Thin-billed prion		Antarctic Prion		Multifactorial ANOVA	
	Males	Females	Males	Females	Males	Females	Species effect	Sex effect
**Pre-laying exodus: N**	4	8	7	8	7	4		
Mean distance to colony (km)	1086 ± 268	2078 ± 826	1429 ± 451	1654 ± 445	813 ± 136	1269 ± 273	***F***_**2,32**_ **= 5.0,*P*** **= 0.013**	***F***_**1,33**_ **= 9.5,*P*** **= 0.004**
Max. distance to colony (km)	1995 ± 510	3418 ± 1283	2225 ± 713	2660 ± 673	1505 ± 317	1842 ± 182	***F***_**2,32**_ **= 5.9,*P*** **< 0.001**	***F***_**1,33**_ **= 6.5,*P*** **= 0.016**
Mean longitude (°E)	71.4 ± 9.0	78.7 ± 29.1	53.0 ± 8.7	47.9 ± 7.9	76.1 ± 4.3	83.1 ± 7.5	***F***_**2,32**_ **= 12.2,*P*** **= 0.002**	*F*_1,33_ = 0.3, *P* = 0.598
Mean latitude (°S)	48.2 ± 3.9	49.4 ± 2.6	52.3 ± 3.2	52.1 ± 1.3	52.0 ± 2.5	55.3 ± 0.7	***F***_**2,32**_ **= 7.8,*P*** **= 0.002**	*F*_1,33_ = 2.8, *P* = 0.101
Time foraging (hours/day)	14.4 ± 1.3	14.4 ± 6.4	17.8 ± 0.6	17.6 ± 0.7	16.1 ± 1.0	14.9 ± 2.1	***F***_**2,32**_ **= 23.0,*P*** **< 0.001**	*F*_1,33_ = 1.5, *P* = 0.236
- During daytime	8.9 ± 0.6	8.4 ± 1.1	12.0 ± 0.5	11.3 ± 0.8	10.3 ± 0.7	9.4 ± 1.5	***F***_**2,32**_ **= 25.4,*P*** **< 0.001**	*F*_1,33_ = 4.1, *P* = 0.052
- During night time	5.1 ± 0.7	5.5 ± 0.6	4.1 ± 0.8	4.0 ± 0.3	3.5 ± 1.1	1.9 ± 0.9	***F***_**2,32**_ **= 26.5,*P*** **< 0.001**	*F*_1,33_ = 2.5, *P* = 0.122
- During dusk	0.8 ± 0.2	0.8 ± 0.1	1.5 ± 0.2	1.4 ± 0.1	2.4 ± 1.0	3.6 ± 1.5	***F***_**2,32**_ **= 24.1,*P*** **< 0.001**	*F*_1,33_ = 2.5, *P* = 0.123
- During dawn	0.2 ± 0.2	0.2 ± 0.2	0.6 ± 0.3	0.9 ± 0.3	0.1 ± 0.2	0.1 ± 0.1	***F***_**2,32**_ **= 19.7,*P*** **< 0.001**	*F*_1,33_ = 0.3, *P* = 0.614
Time in flight (hours/day)	9.1 ± 1.4	8.7 ± 0.4	4.5 ± 0.8	4.0 ± 0.9	6.6 ± 1.4	6.3 ± 3.1	***F***_**2,32**_ **= 24.3,*P*** **< 0.001**	*F*_1,33_ = 0.7, *P* = 0.420
Time on water (hours/day)	0.5 ± 0.2	1.0 ± 0.4	1.7 ± 0.7	2.2 ± 0.4	1.3 ± 0.7	2.8 ± 1.4	***F***_**2,32**_ **= 11.7,*P*** **< 0.001**	***F***_**1,33**_ **= 11.3,*P*** **= 0.002**

Multifactorial ANOVA were carried out with interactions. However, interactions were not statistically significant (all *P* > 0.05) and are therefore not given here

**Fig. 2:**
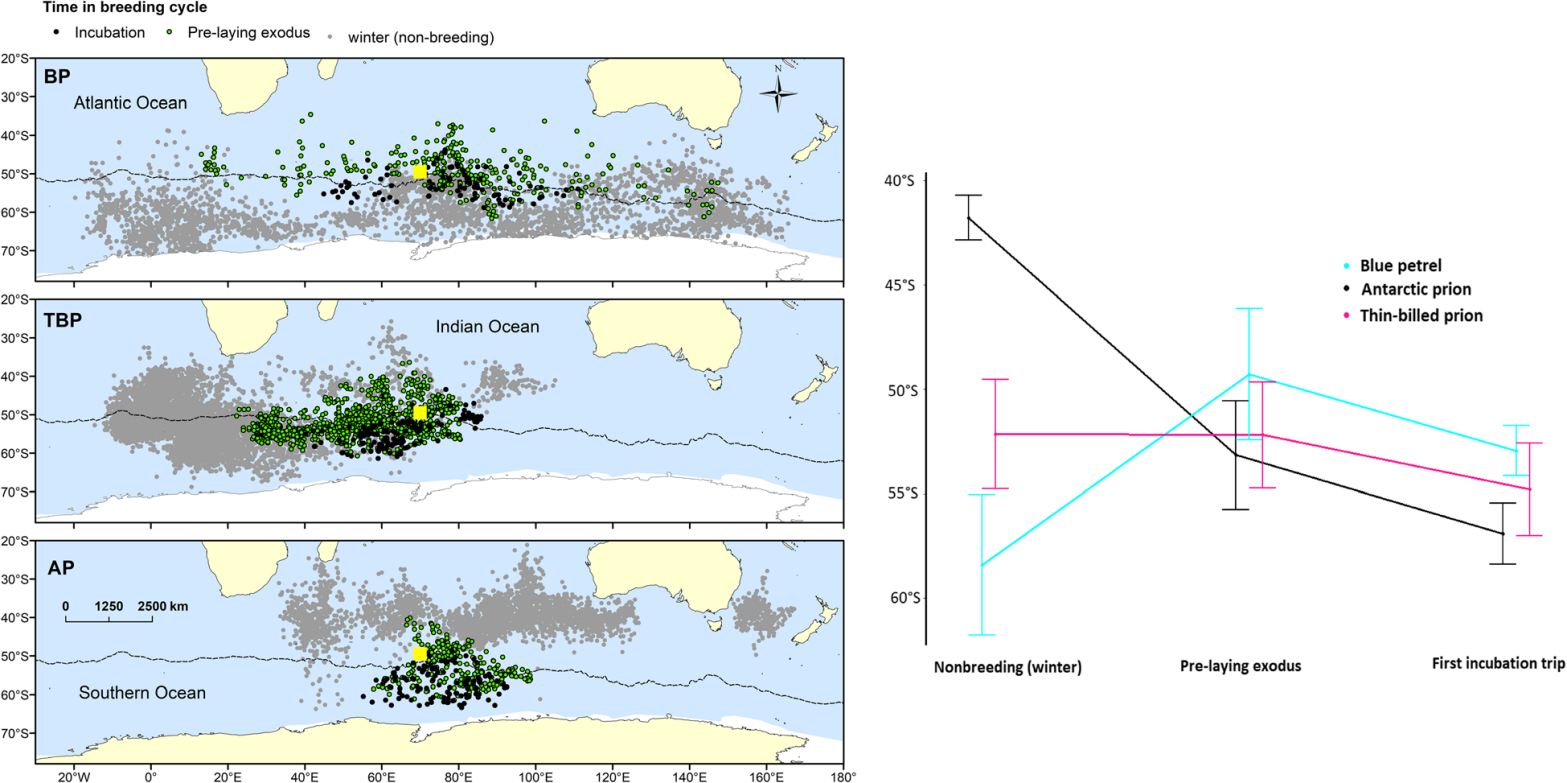
Distribution of Blue Petrels (BP), Thin-billed prions (TBP) and Antarctic Prions (AP) from Kerguelen Islands, during the pre-laying exodus and first incubation trip. The dotted line represents the oceanographic location of the Polar Front. For comparison, the winter distribution is shown in light grey. Right panel: comparison of the latitudinal distribution, showing the leapfrogging of Antarctic prions and Blue petrels

The distribution during the pre-laying exodus also differed among the species (Fig. 2). Blue petrels spread most widely longitudinally, and used more northerly latitudes during the exodus than both Thin-billed and Antarctic prions (Table 4, Fig. 2). Thin-billed prions from Kerguelen mainly used open-ocean waters west of the archipelago, and Antarctic prions moved in an easterly direction (Table 4, Fig. 2), and travelled the shortest distance from the colony (Table 4).

**Fig. 3:**
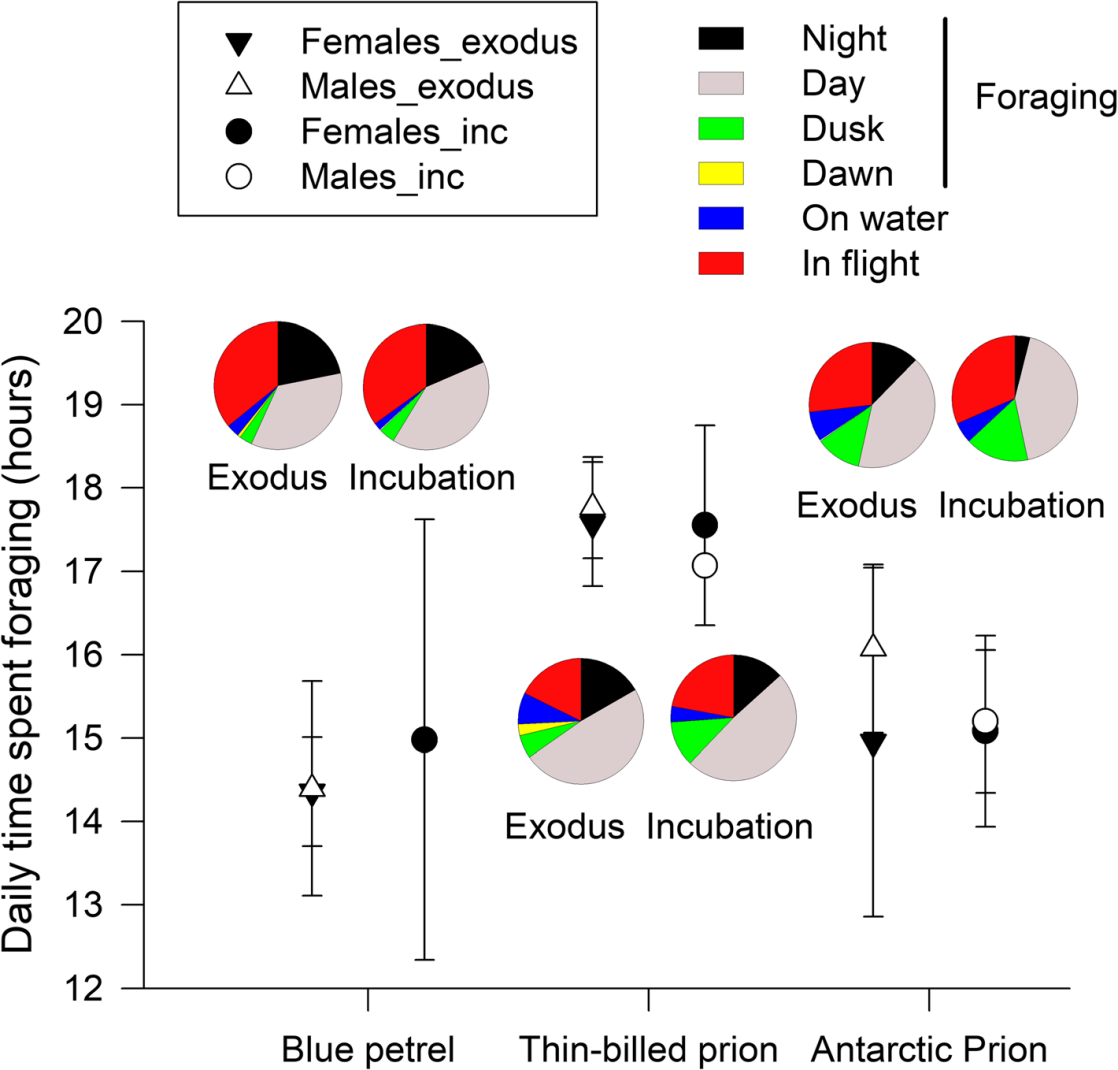
Foraging and flight activities of Blue Petrels, Thin-billed prions and Antarctic Prions from Kerguelen Islands, obtained from immersion loggers

The activity patterns also differed among the species, but not between the sexes (Table 4, Fig. 3). In particular, Blue petrels spent the least time (ca. 14 h) foraging, while Thin-billed prions foraged for nearly 18 h daily, including during about 6 h during the dark hours of night, dusk and dawn (Table 4). In contrast, Blue petrels spent much time in flight (9 h), compared to only 4 h in Thin-billed prions (Fig. 3).

The return dates from the exodus for egg-laying differed among the species, but not between the sexes (Table 2).

### Incubation

The first incubation shifts were taken by the males, and consequently, the first incubation trips were carried out by the females, starting in October in Blue petrels, November in Thin-billed prions and December in Antarctic prions (Fig. 1, Fig. S1, Table 3). We observed species differences in the duration of the first incubation trip (Table 2), with shortest trips in Antarctic prions, intermediate trips in Thin-billed prions and longest trips in Blue petrels (Table 3).

The distribution during the first incubation trip varied latitudinally among the species (Fig. 2), with Blue petrels using more northerly latitudes than Thin-billed prions and Antarctic prions moved into easterly direction (Table 4, Fig. 2), while the distances reached did not differ consistently among the species.

The activity patterns also differed among the species, but not between the sexes (Table 5, Fig. 3). During the incubation trip, Blue petrels and Antarctic prions spent ca. 15 h foraging, while Thin-billed prions foraged for 17–18 h daily. In contrast, Blue petrels spent much time in sustained flight, compared to Thin-billed prions, while flight times were intermediate in Antarctic prions (Fig. 3).

**Table 5:** Distribution and activity parameters of Blue Petrels, Thin-billed prions and Antarctic Prions from Kerguelen Islands during the first incubation trip

	Blue petrel		Thin-billed prion		Antarctic Prion		Multifactorial ANOVA	
	Males	Females	Males	Females	Males	Females	Species effect	Sex effect
**First incubation trip: N**	0	7	1	6	7	4		
Mean distance to colony (km)	–	1158 ± 334	1455	944 ± 40	1067 ± 104	1066 ± 75	*F*_2,32_ = 0.9, *P* = 0.416	*F*_1,32_ = 0.9, *P* = 0.355
Max. distance to colony (km)	–	1840 ± 505	2002	1256 ± 123	1544 ± 163	1608 ± 165	*F*_2,32_ = 2.7, *P* = 0.091	*F*_1,32_ = 0.5, *P* = 0.500
Mean longitude (°E)	–	77.3 ± 13.9	54.0	66.5 ± 9.8	76.1 ± 5.2	74.5 ± 5.6	*F*_2,32_ = 2.9, *P* = 0.082	*F*_1,32_ = 0.1, *P* = 0.753
Mean latitude (°S)	–	52.9 ± 1.2	56.8	54.4 ± 2.2	56.8 ± 1.8	57.0 ± 0.5	***F***_**2,32**_ **= 6.2,*P*** **= 0.009**	*F*_1,32_ = 0.3, *P* = 0.618
Time foraging (hours)	–	15.0 ± 2.6	17.1	17.6 ± 1.2	15.2 ± 0.9	15.1 ± 1.1	***F***_**2,32**_ **= 3.6,*P*** **= 0.048**	*F*_1,32_ < 0.1, *P* = 0.976
- During daytime	–	9.8 ± 1.6	12.9	11.5 ± 0.6	10.2 ± 0.8	10.3 ± 1.1	***F***_**2,32**_ **= 3.9,*P*** **= 0.039**	*F*_1,32_ = 0.2, *P* = 0.672
- During night time	–	4.5 ± 1.0	1.2	3.7 ± 1.8	1.1 ± 0.9	0.7 ± 0.2	***F***_**2,32**_ **= 11.5,*P*** **< 0.001**	*F*_1,32_ = 0.3, *P* = 0.598
- During dusk	–	1.0 ± 0.2	2.9	2.8 ± 1.2	3.8 ± 0.8	4.0 ± 0.5	***F***_**2,32**_ **= 19.8,*P*** **< 0.001**	*F*_1,32_ = 0.1, *P* = 0.750
- During dawn	–	0.1 ± 0.1	0.1	0	0	0	*F*_2,32_ = 2.6, *P* = 0.106	*F*_1,32_ = 0.9, *P* = 0.350
Time in flight (hours)	–	8.5 ± 2.5	6.2	5.2 ± 1.3	7.5 ± 0.8	7.7 ± 1.5	***F***_**2,32**_ **= 5.1,*P*** **= 0.017**	*F*_1,32_ < 0.1, *P* = 0.916
Time on water (hours)	–	0.5 ± 0.2	0.7	1.0 ± 0.6	1.3 ± 0.4	1.2 ± 0.5	***F***_**2,32**_ **= 4.2,*P*** **= 0.032**	*F*_1,32_ < 0.1, *P* = 0.972

Multifactorial ANOVA were carried out with interactions. However, interactions were not statistically significant (all *P* > 0.05) and are therefore not given here

### Distribution and habitat parameters

The latitudinal distribution during the pre-laying exodus differed among the species (ANOVA, *F*_2,35_ = 6.3, *P* = 0.004), and Tukey Post-hoc tests indicated that Blue petrels used more northern latitudes than both Antarctic and Thin-billed prions (Fig. 2).

The latitudinal distribution during the first incubation trip also differed among the species (ANOVA, *F*_2,22_ = 12.7, *P* < 0.001), and Tukey Post-hoc tests here indicated that Antarctic prions used the most southern latitudes, while Blue petrels and Thin-billed prions did not differ statistically significantly (Fig. 2, *P* = 0.075).

Thus, between the winter and breeding seasons, the habitat preferences were reversed, with Antarctic prions changing from northern to southernmost, and Blue petrels in the opposite direction. Thin-billed prions were the most constant species in terms of latitudinal distribution.

**Fig. 4:**
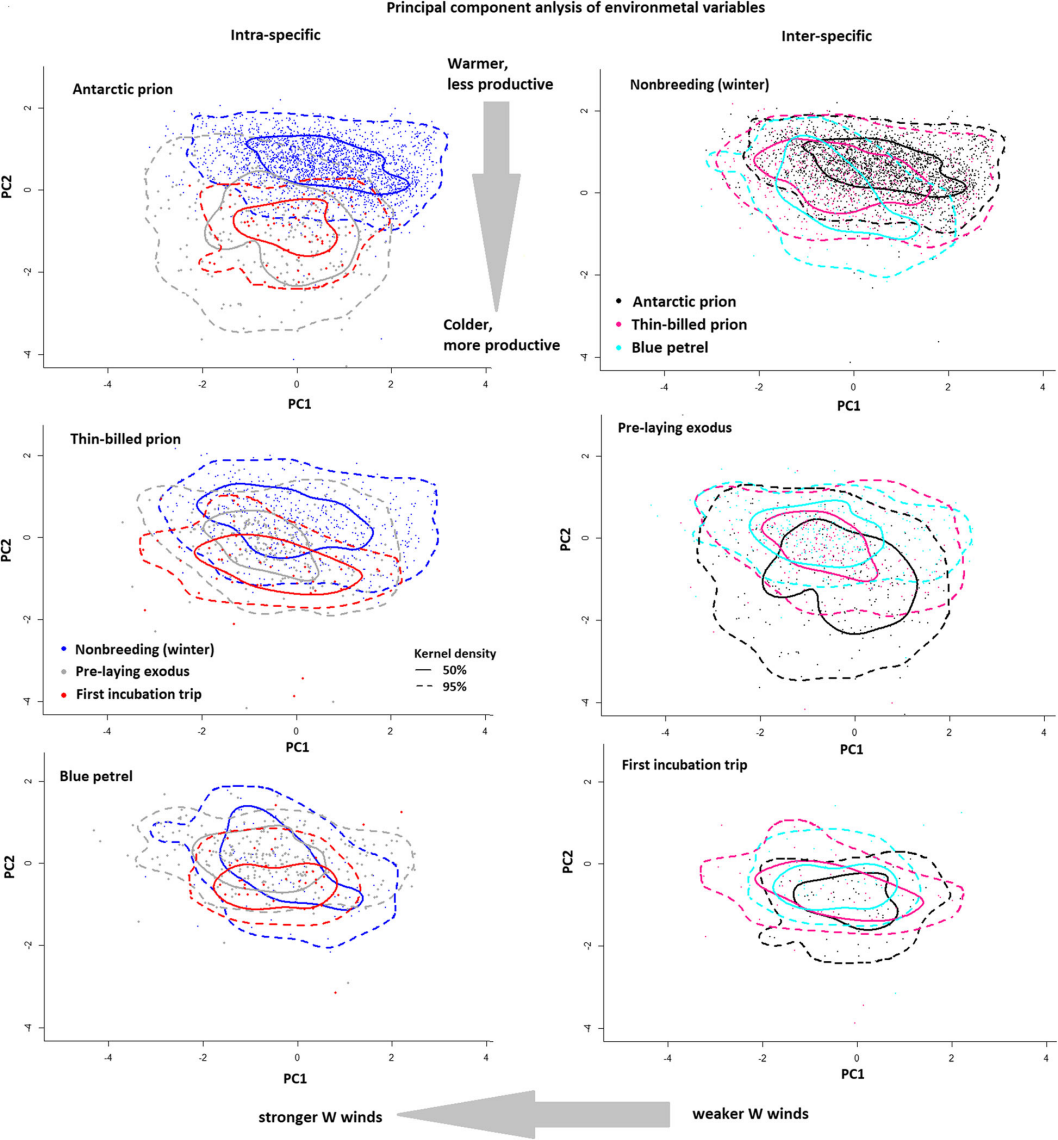
Habitat niches of Blue Petrels, Thin-billed prions and Antarctic Prions from Kerguelen Islands, obtained from distributions and kernel densities of principal component scores of environmental parameters (SST, ChlA, windU, windV, windspeed)

**Fig. 5:**
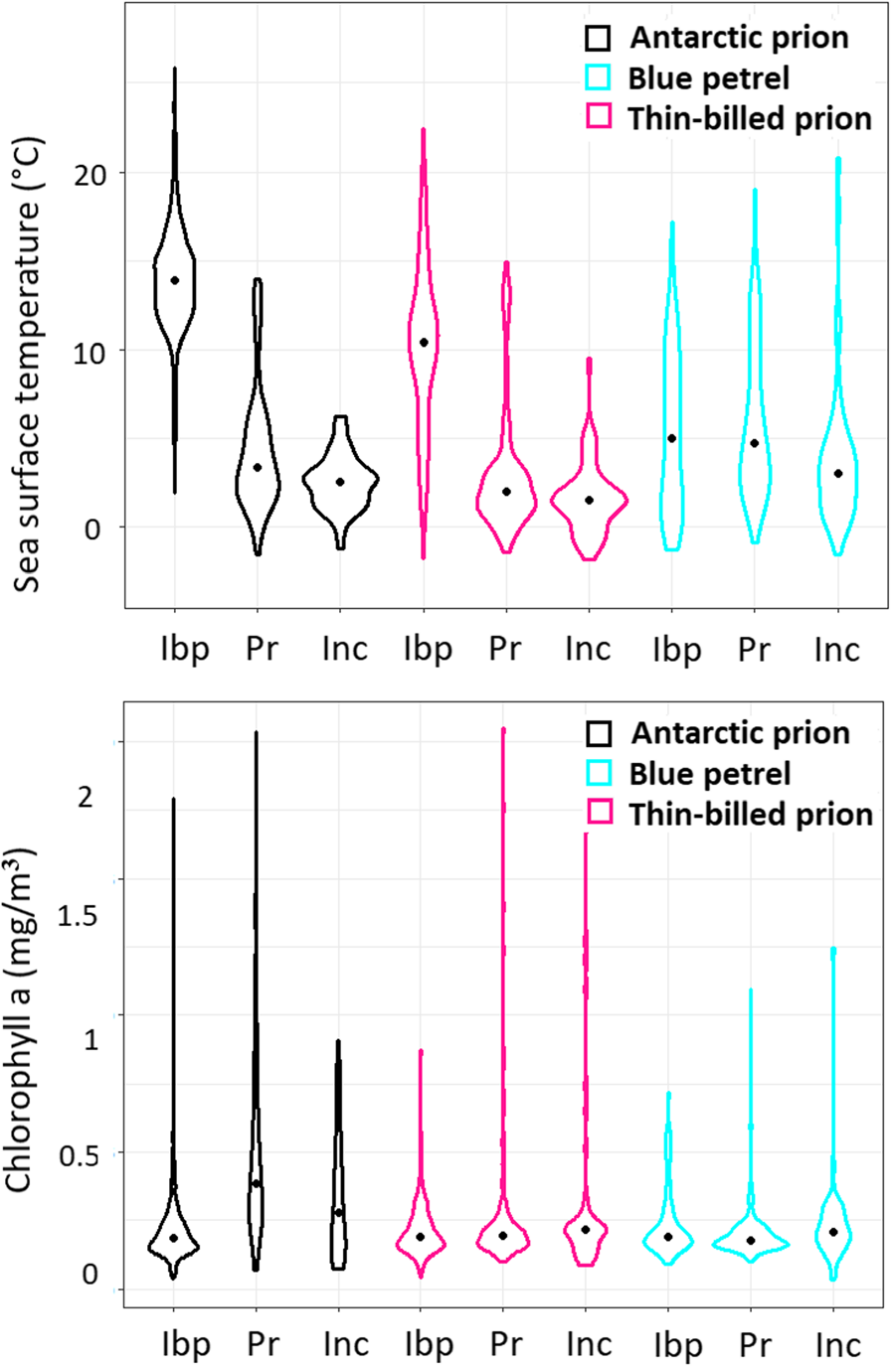
Changes in sea surface temperatures and chlorophyll a in the habitat used by Blue Petrels, Thin-billed prions and Antarctic Prions from Kerguelen Islands, among the phases of the season (Ibp = inter-breeding period, Pr = Pre-laying exodus, Inc. = Incubation)

Given the different distribution and timing during pre-incubation exodus and first incubation trips, this resulted in some differences in the habitat parameters (Table 6). For example, Blue petrels tended to forage in the warmest, low productive waters (Fig. 4), and Antarctic prions experienced the most productive waters (Fig. 5 lower panel) and the calmest conditions with only light winds. When comparing the ecological niches based on these parameters, Antarctic Prions showed a large change between the winter and the breeding season, with 50% kernels of the PCs not overlapping (Fig. 4). In contrast, Blue petrels had largely overlapping seasonal niche spaces, and Thin-billed prions presented an intermediate case. These differences among the species were also seen in some single habitat parameters, especially sea surface temperature (Fig. 5, upper panel).

**Table 6:** Habitat parameters of Blue Petrels, Thin-billed prions and Antarctic Prions from Kerguelen Islands

	Blue petrel		Thin-billed prion		Antarctic Prion	
	Pre-laying exodus	Incubation	Pre-laying exodus	Incubation	Pre-laying exodus	Incubation
Bathymetry (m)	3484 ± 434 ^a^	3723 ± 453 ^a,b^	4271 ± 290 ^b^	4205 ± 509 ^b^	3529 ± 548 ^a^	3251 ± 515 ^a^
Sea surface temperature (°C)	5.3 ± 2.0 ^b^	3.5 ± 1.6 ^a,b^	3.0 ± 2.5 ^a^	1.6 ± 1.4 ^a^	3.7 ± 1.7 ^a,b^	2.4 ± 0.6 ^a^
Chlorophyll a (mg/m^3^)	0.20 ± 0.02 ^a^	0.22 ± 0.03 ^a^	0.25 ± 0.10 ^a^	0.29 ± 0.20 ^a^	0.45 ± 0.09 ^b^	0.30 ± 0.09 ^a^
Wind speed (m/s)	11.4 ± 0.6 ^b^	10.4 ± 1.6 ^b,c^	10.2 ± 1.0 ^c^	10.7 ± 1.3 ^b,c^	9.8 ± 0.9 ^c^	8.5 ± 0.6 ^a^
WindU (west – to east, m/s)	8.9 ± 1.3 ^b^	5.3 ± 2.5 ^c^	7.0 ± 1.5 ^b,c^	7.3 ± 2.4 ^b,c^	4.8 ± 2.6 ^a,c^	2.0 ± 2.7 ^a^
WindV (south to north, m/s)	-1.1 ± 1.1 ^a^	0.1 ± 2.2 ^a,b^	1.3 ± 0.9 ^b^	−2.0 ± 1.6 ^a^	−2.0 ± 0.9 ^a^	−2.0 ± 2.2 ^a,b^

Bathymetry from ECMWF ETOPO1 Elevation, Daytime SST and Chlorophyll a from MODIS Ocean Aqua OceanColor (4 km, 8 day mean), wind from ECMWF Interim Full Daily SFC Wind (10 m above Ground), wind speed was calculated from the square root of the sums of squares of the V and U component. Kruskal-Wallis ANOVAs were carried out for each parameter, to test for differences among the groups. All parameters differed (χ^2^ > 17.9, df = 5, *p* < 0.003), and similar superscript letters denote homogenous subsets

### Stable isotope analyses

**Fig. 6:**
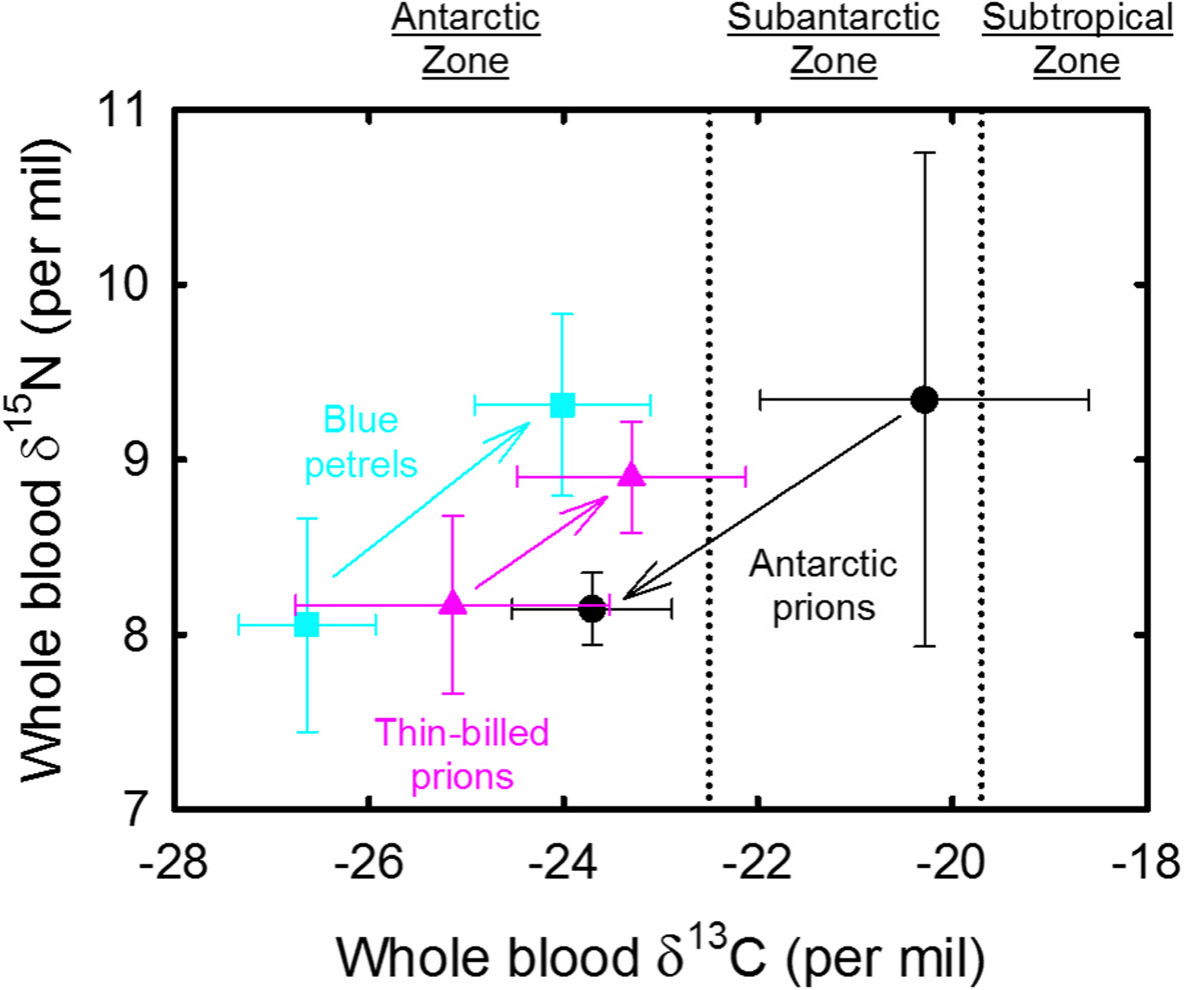
Whole blood δ^15^N versus δ^13^C values of Blue petrels (blue squares), Thin-billed prions (red triangles) and Antarctic prions (black circles) from Kerguelen Islands during the inter-breeding and early breeding periods. Isotopic values of feathers that were moulted during the inter-breeding period were corrected to allow an accurate comparison with blood values (see text). Arrows indicate the isotopic shifts from the inter-breeding to the early breeding period. Values are means ± SD

Isotopic data of whole blood from both sexes were pooled because they were not statistically significant, except δ^15^N values of females and males blue petrels (Mann-Whitney, U = 12.0, *p* = 0.035). In early breeding, blood from the three species were segregated by their stable isotope values (MANOVA, Wilks’s Lambda, *F*_4,92_ = 13.69, *p* < 0.0001), with δ^15^N values, but not δ^13^C values, showing significant variations across species (ANOVA, *F*_2,47_ = 28.95 and 2.38, *p* < 0.0001 and *p* = 0.104, respectively) (Fig. 6, Fig. S2). Blood δ^15^N values increased in the order 8.1 ± 0.2 ‰ (AP, *n* = 10) < 8.9 ± 0.3 ‰ (TBP, *n* = 23) < 9.3 ± 0.5 ‰ (BP, *n* = 17) (post hoc Tukey’s HSD multiple comparison tests, all *p* ≤ 0.005). Interestingly, the five birds with δ^13^C values > − 23.0 ‰ were all males (one AP, two BP and two TBP, Fig. S2).

## Discussion

In the present study, we explored the interplay of differences in timing of migration and the behaviour during the early breeding season. Using bio-logging data we found differences in colony attendance patterns among the three species, determined the foraging areas used during the pre-laying exodus and early incubation, and examined the activity data of tracked birds during the early breeding season. Stable isotopes completed the picture by confirming habitat changes between the inter-breeding and early breeding periods and highlighting resource segregation during the latter.

### Ecological niches

Due to large differences in light and temperature throughout the year, polar environments are strongly seasonal. In marine environments, there is a succession of phytoplankton blooms and zooplankton reproduction and abundance throughout the spring and summer. Seabirds in Polar Regions have evolved different strategies to cope with these seasonal changes. Most polar birds are migratory, and thus move away from the breeding grounds. The three petrel species in this study have been shown to move to different latitudes and thus, environmental conditions, during the winter [13].

Migratory birds can employ either a ‘niche-tracking’ strategy, by moving to habitats with similar environmental conditions, or a ‘niche-switching’ strategy, with changing environmental conditions in the breeding and non-breeding season [39, 40]. While niche segregation within and among seabirds species has received considerable attention lately (e.g. [8, 10, 13]), and other closely-related, sympatric seabird species have also been shown to adopt divergent migratory strategies [41] to our knowledge, the concept of ‘niche-tracking’ vs. ‘niche-switching’ has not been explored in seabirds.

Some care has to be taken in the terminology, as the term ‘niche-tracking’ has lately also been employed to explain shifts of populations in response to climate change [42]. The term ‘niche conservatism’, in contrast, is mostly used to compare niches in evolutionary time [43].

In the present study, Blue petrels spent the winter furthest south and returned earliest to start breeding. Although they used the most northern latitudes during the pre-laying exodus and incubation (Fig. 2), due to the early breeding schedule the water temperatures were still low and spring bloom had not started. Therefore, in terms of the ecological conditions, Blue petrels remained the most constant throughout the year. By breeding early, they were able to track their preferred ecological niche in terms of oceanographic conditions. Comparison of δ^13^C values between body feathers (corrected values) and blood confirmed that pattern with birds remaining within the Antarctic Zone during both the inter-breeding and early breeding periods, but using a more northern habitat during the latter period (Fig. 6).

In contrast, Antarctic prions spent the winter at the most northerly latitudes but during breeding, Antarctic prions used the most southern latitudes, resulting in a niche switch. This habitat switch is highlighted by stable isotopes, with birds showing more positive δ^13^C values during the inter-breeding period than during the early breeding period, meaning a spatial change from subantarctic (subtropical) waters to the Antarctic Zone (Fig. 6). Thin-billed prions were intermediate between the two strategies of niche tracking and niche switching, by remaining most faithful to their latitudinal distribution (Fig. 2) throughout the year. Accordingly, δ^13^C values of Thin-billed prions varied less than those of the two other species. Birds remained within the Antarctic Zone, but foraged at slightly northern latitudes during the early breeding period than during the interbreeding period (Fig. 6).

In songbirds, a common pattern is that of niche-tracking migrants and niche-switching residents [44, 45]. Here, we found a suite of sympatric, closely related migrants using both strategies, and intermediate behaviour. Hypotheses regarding the evolution of migration predict niche-tracking as primitive, and niche-switching as derived [33]. According to this, Antarctic prions would show the most derived state. According to genetic data [14], blue petrels are ancestral, and Thin-billed prions and Antarctic prions are recently (0.9 Ma) derived sister taxa, fitting this pattern.

Another concept that was first developed in terrestrial birds [46, 47], and recently described in seabird ecology, is leapfrog migration, i.e. the case where northerly breeding species or populations migrate longer distances to spend the non-breeding season further south than southerly breeding populations or species. Leapfrog migratory populations have been found in Bulwer’s petrel *Bulweria bulwerii* [48], and the leapfrog concept has also been applied to sympatric Adélie and chinstrap penguins *Pygoscelis adeliae* and *P. antarcticus* [49]. The latter arises where two sympatric, central-place foraging colonial seabirds display a combination of allochrony and stage-dependent foraging ranges [49]. Allochrony is also found in the present study, with mean arrival dates among the species more than 1 month apart (Table 3, Figs. 1 and 2). Allochrony will result in a temporal offset of the peak energetic demands among the three petrels, such as previously shown for chick rearing sympatric Pygoscelid penguins [50] and for Brünnich’s and common guillemots *Uria lomvia* and *U. aalge* [51]. Although the patterns observed here suggest allochrony may result in avoidance of competition, further research may be needed to ascertain if competition is an important selective pressure upon the evolution of Blue petrel and prion phenology and migratory strategies. In contrast to penguins and auks, these petrels are highly mobile, and their early phenology may also have evolved to allow the three species to exploit different peaks in food availability following the spring bloom, and to complete the breeding and moult cycle prior to the onset of the Antarctic winter in species that remain in Antarctic waters (Blue petrels and Thin-billed prions).

### Activity data

During the pre-laying exodus and the incubation trip, Thin-billed prions spent less time in constant flight and more time foraging (17–18 h) than Blue petrels and Antarctic prions (14–15 h, Fig. 3). This was not due to a shorter flight distance, as the maximum distance to the colony was shortest in Antarctic prions during the exodus (Table 4) and did not differ among the species during incubation (Table 5). Thus, differences in the activity patterns are most likely explained by differences in the foraging ecology, i.e. the diet choice or the search and handling time needed to feed on specific prey. The diet of the three species at Kerguelen has been studied in detail during the chick-rearing period [52, 53]. The dietary spectrum partly overlaps, but Blue petrels take considerably more fish (36% by mass) and less crustaceans (61%) than Thin-billed prions (6% fish, 91% crustaceans) and Antarctic prions (5% fish, 89% crustaceans). The most commonly taken crustacean was the hyperiid amphipod *Themisto gaudichaudii*, and the two prions were segregated by feeding on different euphausiids, Thin-billed prions on *Thysanoessa* spp. (16% by mass) and Antarctic prions on *Euphausia vallentini* (15% by mass). Antarctic prions also caught more small prey such as copepods than Thin-billed prions, which is probably related to filtering lamellae present in the beak of Antarctic prions. The diet in the early breeding season is not known, but it is likely that dietary differences would explain differences in the activity patterns observed here. Accordingly, blood δ^15^N values collected at GLS recovery and representing the pre-laying exodus time differed amongst the three species, thus showing trophic segregation. The higher δ^15^N value of Blue petrels is in agreement with the species preying more on mesopelagic fish during the breeding season, thus contrasting with the lower value of Antarctic prions suggesting a crustacean-based diet. Interestingly, the intermediate δ^15^N value of the Thin-billed prion suggests feeding partially on mesopelagic fish in early breeding, thus contrasting with the crustacean diet described during the chick-rearing and inter-breeding periods ([16, 52], Fig. 6).

Both sexes has largely similar daily activity budgets (Table 4). However, females spent more time sitting on the water. This resting time, together with the longer duration of the pre-laying exodus compared to males, may be important for the accumulation of nutrients for the egg.

## Conclusions

We have shown that the three sympatric petrel species segregated in time and space, both in the winter as well as in the early breeding season. The interplay of timing and distribution meant that the three species show the full range of migratory strategies, from niche-tracking Blue petrels to niche-switching Antarctic prions. This meant that the habitat associations were partly reversed, and the latitudinal distribution resembled the leapfrogging of terrestrial avian migrant species or populations. More information on the diet taken during the different stages of the breeding cycle would be highly informative in order to explain differences in phenology and activity patterns, as the three species most likely exploit different peaks in food availability.

## Supplementary information

**Additional file 1: Figure S1.** Individual early breeding season chronologies of Blue Petrels (BP), Thin-billed prions (TBP) and Antarctic Prions (AP) from Kerguelen Islands. Color bars show the timing and duration of the subsequent phases from arrival to the colony (first yellow block) until recapture (marked with #). Pre-breeding times at sea are marked in turquoise, and the moult of flight fathers in Antarctic prions is marked in grey. Colony attendance before the prelaying exodus consisted of shifts of in the burrow (in yellow), followed by the pre-laying exodus (in pink). After return from the pre-laying exodus, the birds spent 1–16 days in the burrow (in yellow), followed by a first foraging trips (marked in green).

**Additional file 2: Figure S2.** Whole blood δ^15^N versus δ^13^C values of Blue petrels (blue squares), Thin-billed prions (red triangles) and Antarctic prions (black circles) from Kerguelen Islands during the early breeding period. Both individual values and means ± SD are indicated.

## Data Availability

The datasets supporting the conclusions of this article are available in the MOVEBANK repository.

## Abbreviations

ANOVA: Analysis of Variance
AP: Antarctic prions (*Pachyptila desolata*)
BP: Blue petrels (*Halobaena caerulea*)
ChlA: Chlorophyll a mass concentration
Env-DATA: Environmental Data Automated Track Annotation System on Movebank (movebank.org)
PCA: Principal component analysis
SST: Sea surface temperature
TBP: Thin-billed prions (*Pachyptila belcheri*)
windU: U (west to east) wind component
windV: V (south to north) wind component

## References

[1] Ainley DG, Dugger KD, Ford RG, Pierce SD, Reese DC, Brodeur RD, Tynan CT, Barth JA (2009). Association of predators and prey at frontal features in the California current: competition, facilitation, and co-occurrence. Mar Ecol Prog Ser.

[2] Henkel LA (2009). Co-occurrence and aggregation of marine birds and mammals in Monterey Bay, California, USA. Mar Ecol Prog Ser.

[3] Pianka ER (1969). Sympatry of desert lizards, and spatial heterogeneity. Ecology.

[4] MacArthur RH (1958). Population ecology of some warblers of northeastern coniferous forests. Ecology.

[5] Hutchinson GE (1959). Homage to Santa Rosalia, or why are there so many different kinds of animals?. Am Nat.

[6] Holt RD (2009). Bringing the Hutchinsonian niche into the 21st century: ecological and evolutionary perspectives. Proc Natl Acad Sci U S A.

[7] Schoener TW (1974). Resource partitioning in ecological communities. Science.

[8] Navarro J, Forero MG, Gonzalez-Solis J, Igual JM, Becares J, Hobson KA (2009). Foraging segregation between two closely related shearwaters breeding in sympatry. Biol Lett.

[9] Wilson RP (2010). Resource partitioning and niche hyper-volume overlap in free-living Pygoscelid penguins. Funct Ecol.

[10] Masello JF, Mundry R, Poisbleau M, Demongin L, Voigt CC, Wikelski M, Quillfeldt P (2010). Diving seabirds share foraging space and time within and among species. Ecosphere.

[11] Ridoux V (1994). The diets and dietary segregation of seabirds at the subantarctic Crozet Islands. Mar Ornithol.

[12] Weiss F, Furness RW, McGill RAR, Strange IJ, Masello JF, Quillfeldt P (2009). Trophic segregation of Falkland Islands seabirds: insights from stable isotope analysis. Polar Biol.

[13] Quillfeldt P, Cherel Y, Delord K, Weimerkirch H (2015). Cool, cold or colder? Spatial segregation of prions and blue petrels is explained by differences in preferred sea surface temperatures. Biol Lett.

[14] Masello JF, Quillfeldt P, Sandoval-Castellanos E, Alderman R, Calderón L, Cherel Y, Cole TL, Cuthbert RJ, Marin M, Massaro M, Navarro J, Phillips RA, Ryan PG, Shepherd LD, Suazo CG, Weimerskirch H, Moodley Y (2019). Additive traits lead to feeding advantage and reproductive isolation, promoting homoploid hybrid speciation. Mol Biol Evol.

[15] Cherel Y, Quillfeldt P, Delord K, Weimerskirch H (2016). Combination of at-sea activity, geolocation and feather stable isotopes documents where and when seabirds molt. Front Ecol Evol.

[16] Cherel Y, Connan M, Jaeger A, Richard P (2014). Seabird year-round and historical feeding ecology: blood and feather δ^13^C and δ^15^N values document foraging plasticity of small sympatric petrels. Mar Ecol Prog Ser.

[17] Quillfeldt P, McGill RAR, Furness RW, Möstl E, Ludynia K, Masello JF (2012). Impact of miniature geolocation loggers on a small petrel, the thin-billed prion *Pachyptila belcheri*. Mar Biol.

[18] Fugler SR, Hunter S, Newton IP, Steele WK (1987). Breeding biology of blue petrels *Halobaena caerulea* at the Prince Edward Islands. Emu.

[19] Marchant S, Higgins PJ (1990). Handbook of Australian, New Zealand and Antarctic birds.

[20] Weimerskirch H, Zotier R, Jouventin P (1989). The avifauna of the Kerguelen Islands. Emu.

[21] Brooke M d L (2004). Albatrosses and petrels across the world.

[22] Warham J (1990). The petrels: their ecology and breeding systems.

[23] Fridolfsson AK, Ellegren H (1999). A simple and universal method for molecular sexing of non-ratite birds. J Avian Biol.

[24] Phillips RA, Silk JRD, Croxall JP, Afanasyev V, Briggs DR (2004). Accuracy of geolocation estimates for flying seabirds. Mar Ecol Prog Ser.

[25] Guilford T, Wynn R, McMinn M, Rodríguez A, Fayet A, Maurice L (2012). Geolocators reveal migration and pre-breeding behaviour of the critically endangered Balearic shearwater *Puffinus mauretanicus*. PLoS One.

[26] Quillfeldt P, Phillips RA, Marx M, Masello JF (2014). Colony attendance and at-sea distribution of thin-billed prions during the early breeding season. J Avian Biol.

[27] Schreiber EA, Burger J (2001). Biology of marine birds.

[28] Mattern T, Masello JF, Ellenberg U, Quillfeldt P (2015). Actave.net - a web-based tool for the analysis of seabird activity patterns from saltwater immersion geolocators. Methods Ecol Evol.

[29] McKnight A, Irons DB, Allyn AJ, Sullivan KM, Suryan RM (2011). Winter dispersal and activity patterns of post-breeding black-legged kittiwakes *Rissa tridactyla* from Prince William Sound, Alaska. Mar Ecol Prog Ser.

[30] Quillfeldt P, Engler JO, Silk JR, Phillips RA (2017). Influence of device accuracy and choice of algorithm for species distribution modelling of seabirds: a case study using black-browed albatrosses. J Avian Biol.

[31] R Development Core Team (2018). R: a language and environment for statistical computing.

[32] Peterson AT (2007). Why not WhyWhere: the need for more complex models of simpler environmental spaces. Ecol Model.

[33] Cherel Y, Hobson KA (2007). Geographical variation in carbon stable isotope signatures of marine predators: a tool to investigate their foraging areas in the Southern Ocean. Mar Ecol Prog Ser.

[34] Quillfeldt P, Voigt CC, Masello JF (2010). Plasticity versus repeatability in seabird migratory behaviour. Behav Ecol Sociobiol.

[35] Cherel Y, Fontaine C, Richard P, Labat JP (2010). Isotopic niches and trophic levels of myctophid fishes and their predators in the Southern Ocean. Limnol Oceanogr.

[36] Cherel Y, Hobson KA, Bailleul F, Groscolas R (2005). Nutrition, physiology, and stable isotopes: new information from fasting and molting penguins. Ecology.

[37] Hobson KA, Clark RG (1992). Assessing avian diets using stable isotopes I: turnover of ^13^C in tissues. Condor.

[38] Cherel Y, Jaquemet S, Maglio A, Jaeger A (2014). Differences in δ^13^C and δ^15^N values between feathers and blood of seabird chicks: implications for non-invasive isotopic investigations. Mar Biol.

[39] Martínez-Meyer E, Townsend Peterson A, Navarro-Sigüenza AG (2004). Evolution of seasonal ecological niches in the *Passerina* buntings (Aves: Cardinalidae). Proc R Soc Lond B Biol Sci.

[40] Nakazawa Y, Peterson AT, Martínez-Meyer E, Navarro-Sigüenza AG (2004). Seasonal niches of Nearctic-Neotropical migratory birds: implications for the evolution of migration. Auk.

[41] Fort J, Steen H, Strøm H, Tremblay Y, Grønningsæter E, Pettex E, Grémillet D (2013). Energetic consequences of contrasting winter migratory strategies in a sympatric Arctic seabird duet. J Avian Biol.

[42] Enriquez-Urzelai U, Bernardo N, Moreno-Rueda G, Montori A, Llorente G (2019). Are amphibians tracking their climatic niches in response to climate warming? A test with Iberian amphibians. Clim Chang.

[43] Peterson AT, Soberón J, Sánchez-Cordero V (1999). Conservatism of ecological niches in evolutionary time. Science.

[44] Gómez C, Tenorio EA, Montoya P, Cadena CD (2016). Niche-tracking migrants and niche-switching residents: evolution of climatic niches in New World warblers (Parulidae). Proc R Soc B.

[45] Fandos Guillermo, Tellería José Luis (2020). Seasonal niche‐tracking behaviour of two partially migratory passerines. Ibis.

[46] Salomonsen F (1955). The evolutionary significance of bird migration. Danske Biologiske Meddelelser.

[47] Alerstam T, Hogstedt G (1980). Spring predictability and leap-frog migration. Ornis Scand.

[48] Ramos R, Sanz V, Militão T, Bried J, Neves VC, Biscoito M, González-Solís J (2015). Leapfrog migration and habitat preferences of a small oceanic seabird, Bulwer’s petrel (*Bulweria bulwerii*). J Biogeogr.

[49] Clewlow HL, Takahashi A, Watanabe S, Votier SC, Downie R, Ratcliffe N (2019). Niche partitioning of sympatric penguins by leapfrog foraging appears to be resilient to climate change. J Anim Ecol.

[50] Trivelpiece WZ, Trivelpiece SG, Volkman NJ (1987). Ecological segregation of Adelie, Gentoo, and Chinstrap penguins at King George Island, Antarctica. Ecology.

[51] Barrett RT, Asheim M, Bakken V (1997). Ecological relationships between two sympatric congeneric species, Common Murres and Thick-billed Murres, *Uria aalge* and *U. lomvia*, breeding in the Barents Sea. Can J Zool.

[52] Cherel Y, Bocher P, De Broyer C, Hobson KA (2002). Food and feeding ecology of the sympatric thin-billed *Pachyptila belcheri* and Antarctic *P. desolata* prions at Iles Kerguelen, Southern Indian Ocean. Mar Ecol Prog Ser.

[53] Cherel Y, Bocher P, Trouvé C, Weimerskirch H (2002). Diet and feeding ecology of blue petrels *Halobaena caerulea* at Iles Kerguelen, Southern Indian Ocean. Mar Ecol Prog Ser.

