# Supplementary material for: Niche switching and leapfrog foraging: movement ecology of sympatric petrels during the early breeding season
Source: Mov Ecol. 2020 May 29;8:23. doi: 10.1186/s40462-020-00212-y (PMC7260822; doi:10.1186/s40462-020-00212-y)
Supplement: Supplementary file 1 — Additional file 1: Figure S1. Individual early breeding season chronologies of Blue Petrels (BP), Thin-billed prions (TBP) and Antarctic Prions (AP) from Kerguelen Islands. Color bars show the timing and duration of the subsequent phases from arrival to the colony (first yellow block) until recapture (marked with #). Pre-breeding times at sea are marked in turquoise, and the moult of flight fathers in Antarctic prions is marked in grey. Colony attendance before the prelaying exodus consisted of shifts of in the burrow (in yellow), followed by the pre-laying exodus (in pink). After return from the pre-laying exodus, the birds spent 1–16 days in the burrow (in yellow), followed by a first foraging trips (marked in green). [file 40462_2020_212_MOESM1_ESM.pdf]

Niche switching and leapfrog foraging: movement ecology of sympatric petrels during the early breeding season

Petra Quillfeldt, Henri Weimerskirch, Karine Delord, Yves Cherel

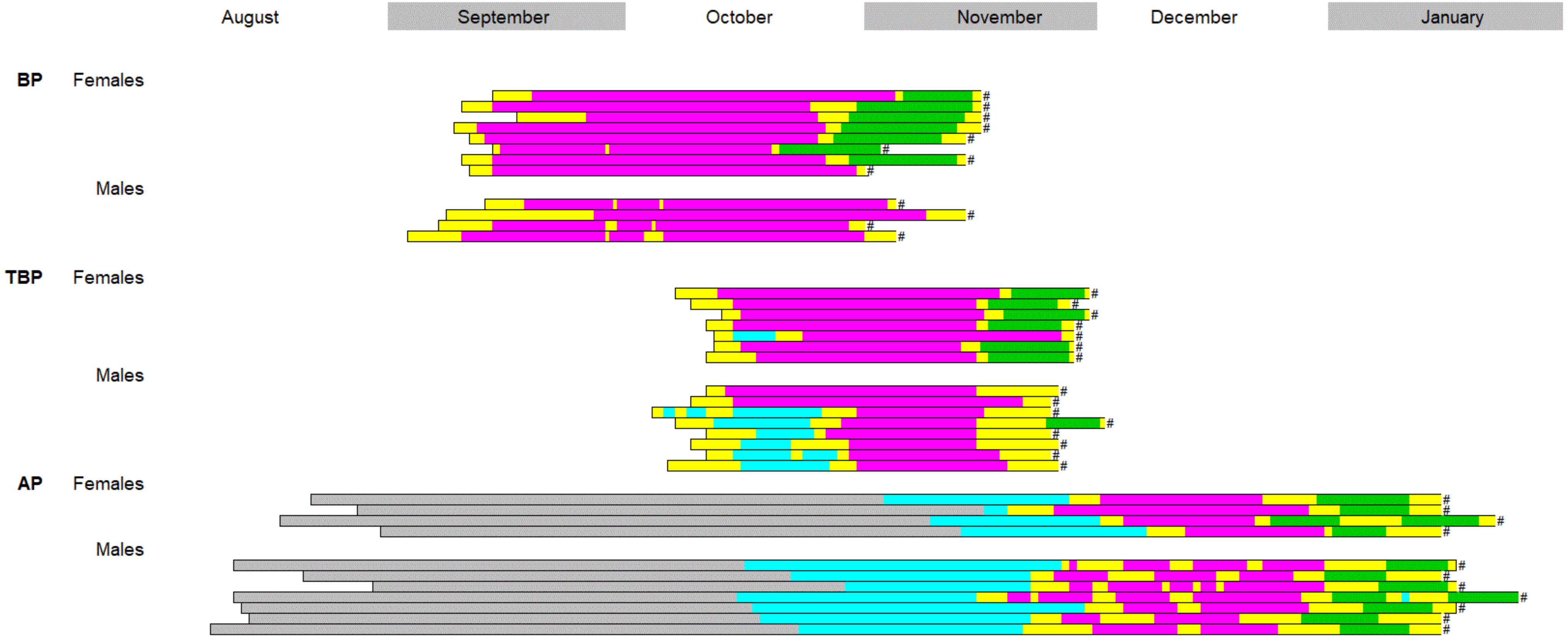

Fig. S1. Individual early breeding season chronologies of Blue Petrels (BP), Thin-billed prions (TBP) and Antarctic Prions (AP) from Kerguelen Islands. Color bars show the timing and duration of the subsequent phases from arrival to the colony (first yellow block) until recapture (marked with #). Pre-breeding times at sea are marked in turquoise, and the moult of flight fathers in Antarctic prions is marked in grey. Colony attendance before the pre-laying exodus consisted of shifts of in the burrow (in yellow), followed by the pre-laying exodus (in pink). After return from the pre-laying exodus, the birds spent 1–16 days in the burrow (in yellow), followed by a first foraging trips (marked in green).
