# Supplementary material for: Niche switching and leapfrog foraging: movement ecology of sympatric petrels during the early breeding season
Source: Mov Ecol. 2020 May 29;8:23. doi: 10.1186/s40462-020-00212-y (PMC7260822; doi:10.1186/s40462-020-00212-y)
Supplement: Supplementary file 2 — Additional file 2: Figure S2. Whole blood δ15N versus δ13C values of Blue petrels (blue squares), Thin-billed prions (red triangles) and Antarctic prions (black circles) from Kerguelen Islands during the early breeding period. Both individual values and means ± SD are indicated. [file 40462_2020_212_MOESM2_ESM.pdf]

Niche switching and leapfrog foraging: movement ecology of sympatric petrels during the early breeding season

Petra Quillfeldt, Henri Weimerskirch, Karine Delord, Yves Cherel

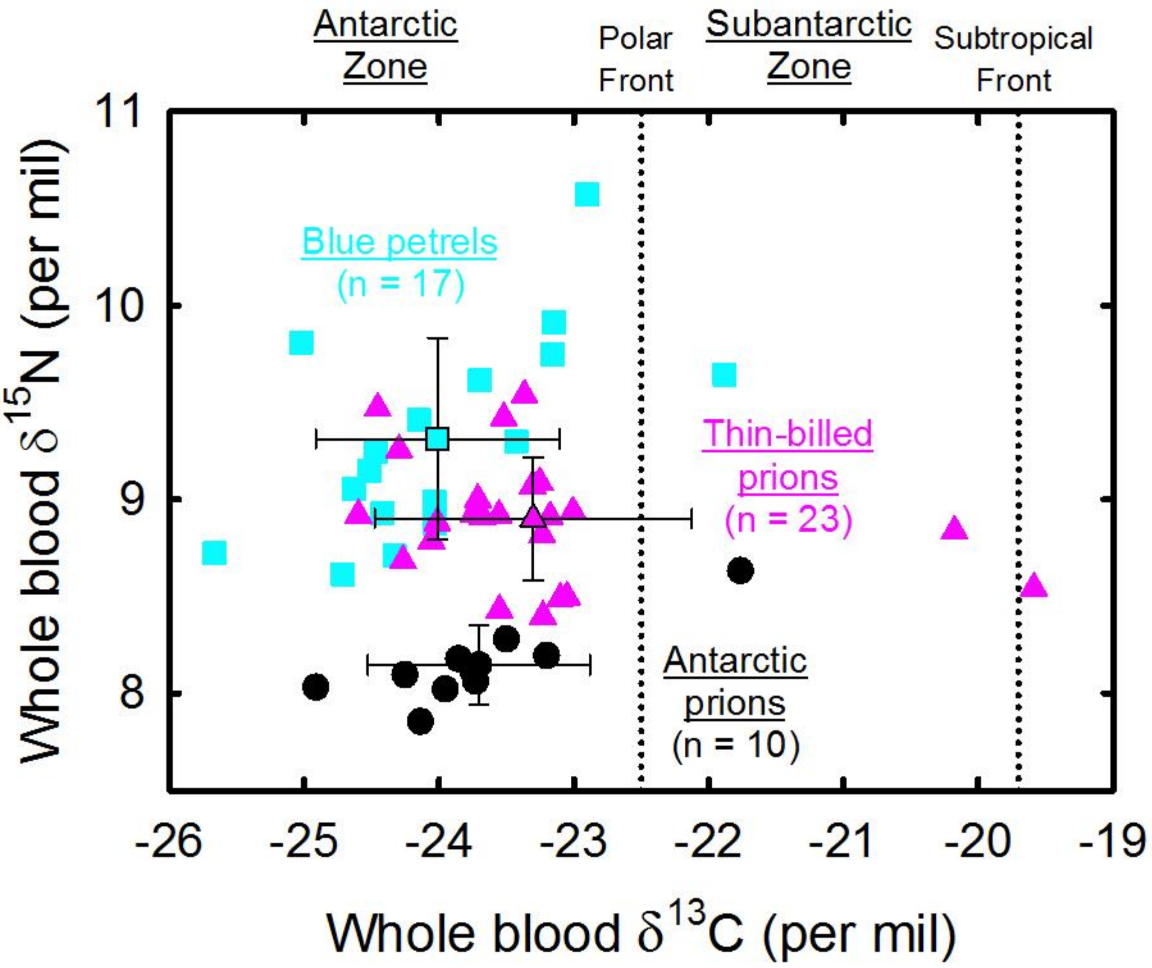

Fig. S2. Whole blood δ^{15}N versus δ^{13}C values of Blue petrels (blue squares), Thin-billed prions (red triangles) and Antarctic prions (black circles) from Kerguelen Islands during the early breeding period. Both individual values and means ± SD are indicated.
